# Oocyte Competence, Embryological Outcomes and miRNA Signature of Different Sized Follicles from Poor Responder Patients

**DOI:** 10.3390/ijms251910237

**Published:** 2024-09-24

**Authors:** Roberto Yagüe-Serrano, Andrea Palomar, Alicia Quiñonero, Víctor Hugo Gómez, Maria José de los Santos, Carmen Vidal, Francisco Dominguez

**Affiliations:** 1 IVIRMA Global Research Alliance, IVI Foundation, Instituto de Investigación Sanitaria La Fe (IIS La Fe), 46026 Valencia, Spain; roberto.yague@ivirma.com (R.Y.-S.); andrea.palomar@ivirma.com (A.P.); alicia.quinonero@ivirma.com (A.Q.); 2 IVIRMA Global Research Alliance, IVIRMA Valencia, 46026 Valencia, Spain; victorhugo.gomez@ivirma.com (V.H.G.); mariajose.delossantos@ivirma.com (M.J.d.l.S.); carmina.vidal@ivirma.com (C.V.)

**Keywords:** poor ovarian response, ovarian follicle, small size follicle, oocyte maturation, oocyte competence, miRNA, molecular marker

## Abstract

Poor ovarian response (POR) patients often face the risk of not having enough competent oocytes. Then, aspirating small follicles could serve as a strategy to increase their number. Many efforts have been addressed to associate follicular size with oocyte competence, but results are controversial. Therefore, our study aimed to evaluate oocyte maturation and developmental competence, along with a non-invasive oocyte-maturation-related miRNA signature in oocytes retrieved from both large and small follicles. A total of 178 follicles, from 31 POR patients, were aspirated and measured on the day of ovarian puncture. Follicular diameters, oocyte collection, oocyte maturation, fertilization, blastocysts, and good-quality blastocyst rates were recorded. Simultaneously, follicular fluids were collected to quantify their miRNA expression. The efficacy of oocyte retrieval along with oocyte maturation, fertilization, and blastulation rates tended to increase with follicular size, but few significant differences were found. Despite there being significantly more collected oocytes from follicles > 11.5 mm compared to follicles ≤ 11.5 mm (*p* < 0.05), oocytes from the latter were also mature, with no significant differences in the miRNA signature, but only those > 13.5 mm demonstrated developmental competence**.** In conclusion, 11.5 mm follicles can produce mature oocytes, but only those larger than 13.5 mm yielded transferable embryos.

## 1. Introduction

Poor ovarian response (POR) affects approximately 9–24% of patients undergoing controlled ovarian stimulation (COS) for in vitro fertilization (IVF) and conceals poor reproductive prognoses such as reduced number of oocytes retrieved and lower pregnancy rates in comparison with normal responder patients [1,2,3].

The wide variation in reported prevalence of poor ovarian response (POR) can be attributed to the lack of consensus and unified criteria for defining POR, which leads to these patients being characterized as a heterogeneous population [4]. Currently, the POSEIDON criteria provide the most standardized approach, offering a better stratification of “low prognosis patients” into four subgroups: Group 1 and Group 2, termed unexpected low prognosis, and Group 3 and Group 4, termed expected low prognosis. This classification is based on ovarian reserve biomarkers [antral follicle count (AFC) and anti-Müllerian hormone (AMH)], previous ovarian response, quantitative and qualitative parameters (such as age and expected aneuploidy rate), and the number of oocytes required for a specific patient to obtain at least one euploid embryo for transfer [5].

The number of retrieved oocytes is especially critical for these patients, making it essential to find ways to increase this number, such as by utilizing oocytes derived from small follicles, for effective treatment.

While follicular size has been linked with oocyte competence and IVF outcomes [6,7,8,9,10,11,12,13,14,15,16,17,18], the likelihood of retrieving competent oocytes not only exclusively from large follicles but also from small follicles should not be dismissed. Smaller follicles were associated with limited oocyte recovery [6,11,12,15,19], immature oocytes [10,13,17,20,21], lower fertilization [11,12,14,15,21,22,23], cleavage rates [10,11,18], and limited embryo quality [12,15,17]. However, small follicles have also demonstrated to be capable of sustaining normal oocyte development [7,9,19,24,25,26] and acceptable embryo quality for either transfer or cryopreservation [7,8,10,12,19,23,25]. The discrepancies among these opposing results may be due to differences in the assessment of follicular size [i.e., measurement of FF volume [14,24] or follicular diameter [12,19]; ranges of follicular sizes, patient cohorts, or other reproductive variables]. Notably, prior to 2000, the outcomes of IVF or intracytoplasmic sperm injection (ICSI) were compared considering oocytes derived from both small and large follicles [11,13,22,27,28]. Differences in fertilization and pregnancy rates between these techniques was probably due to the conventional IVF group containing a larger proportion of immature oocytes derived from small follicles, which are not usually included for ICSI [13]. Further, the patients included in these studies were couples undergoing COS and IVF with different infertility causes [12,17,18].

Micro RNAs (miRNAs) are small non-coding RNA sequences (18–22 nucleotides) that mediate post-transcriptional gene expression [29]. MiRNAs have become reliable biomarkers across different medical specialties [30] as they are tissue-specific master regulators of diverse biological processes, including proliferation, differentiation, migration, and apoptosis [31,32]. The tight coordination of these processes during folliculogenesis is necessary to ensure the oocytes obtain sufficient nutrients and regulatory signals to promote the oocytes’ nuclear and cytoplasmic competence [33]. Aberrant miRNA regulation has been described in other fertility-related pathologies such as endometriosis, endometrial cancer, ectopic pregnancy, and preeclampsia [31,34,35,36,37], but its role in small follicles remains unclear. MiRNA expression was previously reported in ovarian tissue [38,39] and related to oocyte maturation [40]. Indeed, a previous study from our group identified a miRNA maturation signature present in the FF of IVF patients [41]. Consequently, a signature of miRNA expression associated to oocyte maturation will support classical assessment, which is mainly morphology-based and would be especially valuable from a clinical standpoint.

Thus, the main objective of this study is to assess the relationship between follicular size at the time of oocyte retrieval and IVF outcomes (particularly the oocyte collection and quality) in patients with POR. To ascertain whether smaller antral follicles could produce mature and competent oocytes for clinical use, we compared embryology outcomes of the oocytes obtained from a range of follicular sizes and validated oocyte maturation using non-invasive miRNA biomarkers.

## 2. Results

### 2.1. Baseline Reproductive Characteristics

The overall baseline reproductive characteristics of the 31 participants are presented in Table 1. Patients had a median age of 37 years [IQR: 35, 38] and a mean body mass index (BMI) of 22.4 kg/m^2^. Median [IQR] of anti-müllerian hormone (AMH) levels below 1.2 ng/mL support the POR diagnosis. Most included patients belong to Group 4 according to POSEIDON criteria because of their age (≥35 years) [5]. Regarding the rest of reproductive characteristics, stimulation time spanned a mean of 11.28 ± 2.865 days, and total mean gonadotropin dose used was 2042 ± 957.1 IU. On trigger day, the mean of serum estradiol (E2) concentration was 952.3 ± 392.3 pg/mL, whereas serum progesterone (P4) concentration was 0.583 ± 0.378 ng/mL.

### 2.2. Follicles Larger than 11.5 mm Had Superior Embryology Outcomes

The punctured follicles (n = 178) were classified into seven groups according to their size. The embryology outcomes reported as relativized to both the number of punctured follicles and recovered oocytes of each group are presented in Table 2.

Overall, among the 104 oocytes recovered from 178 aspirated follicles (58.43% collection efficacy), there were 80 metaphase II (MII) oocytes (44.94% per punctured follicle or 76.92% per oocyte), with 50 of the MII oocytes recovered being normally fertilized (28.09% per punctured follicle or 62.50% per oocyte). Out of 50 zygotes, 24 of them finally developed into blastocysts (13.48% per punctured follicle or 48% per fertilized oocyte), of which 12 were considered good-quality blastocysts (6.74% per punctured follicle or 24% per fertilized oocyte). Noteworthily, calculated rates considering punctured follicles as a unit of analysis provide anticipated information about embryological outcomes of each follicle before starting IVF procedures.

**Figure 1:**
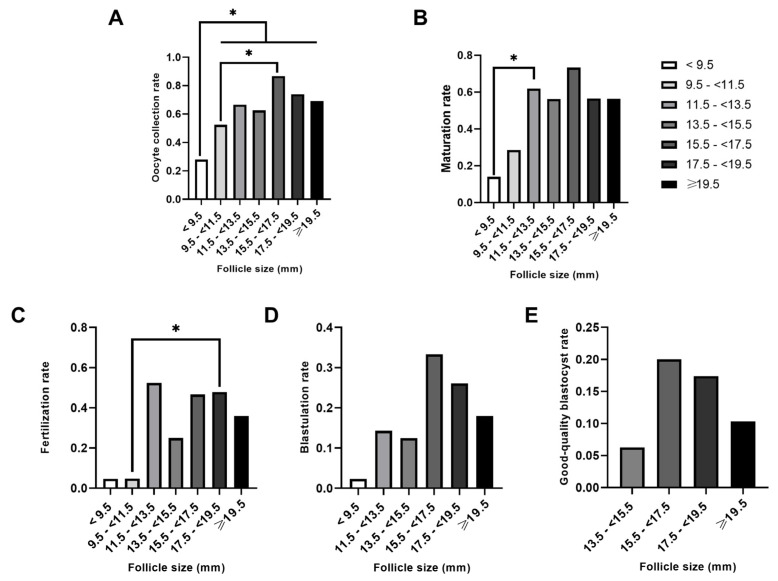
Comparison of the oocyte and embryology outcomes by follicle groups. Follicles were classified into seven groups according to their size (diameter). Rates were calculated based on the number of punctured follicles: (**A**) Oocyte collection rate, (**B**) Maturation rate, (**C**) Fertilization rate, (**D**) Blastulation rate, (**E**) Good-quality Blastocyst rare. * *p* < 0.05. An absence of an asterisk (*) indicates a lack of significant differences. Groups with rates of zero are not shown.

Our results supported the idea that embryology outcomes improved alongside follicular size (Table 2, Figure 1). Rates of oocyte collection ranged from 27.10% in follicles < 9.5 mm to 87.50% in follicles 15.5*–*<17.5 mm in size on the day of oocyte retrieval (Figure 1A, Table 2). Significantly, the number of retrieved oocytes increased when follicles were sized >11.5 mm compared to the smallest follicles (<9.5 mm; *p* < 0.03 in each case; Figure 1A). A similar trend was observed when larger follicles (sized between 15.5 and <17.5 mm) were compared to follicles sized between 9.5 and <11.5 mm (*p* < 0.04) (Figure 1A). In terms of oocyte maturation rates, significant differences were only found between follicles sized < 9.5 mm and 11.5*–*<13.5 mm (*p* = 0.02; Figure 1B). Oocytes from follicles sized between 17.5 and <19.5 mm were associated with significant increase in fertilization rates when compared with oocytes from follicles sized less than 11.5 mm (9.5*–*<11.5 mm; *p* < 0.03; Figure 1C). Only one blastocyst was obtained from all follicles ≤ 11.5 mm in size, while the remaining 23 blastocysts were derived from follicles ≥ 11.5 mm (Figure 1D, Table 2). No follicles ≤ 11.5 mm produced good-quality blastocysts (Figure 1E, Table 2). In both cases, the blastocyst rate (Figure 1D) and the good-quality blastocyst rate (Figure 1E) did not vary significantly among the different follicle size groups.

**Figure 2:**
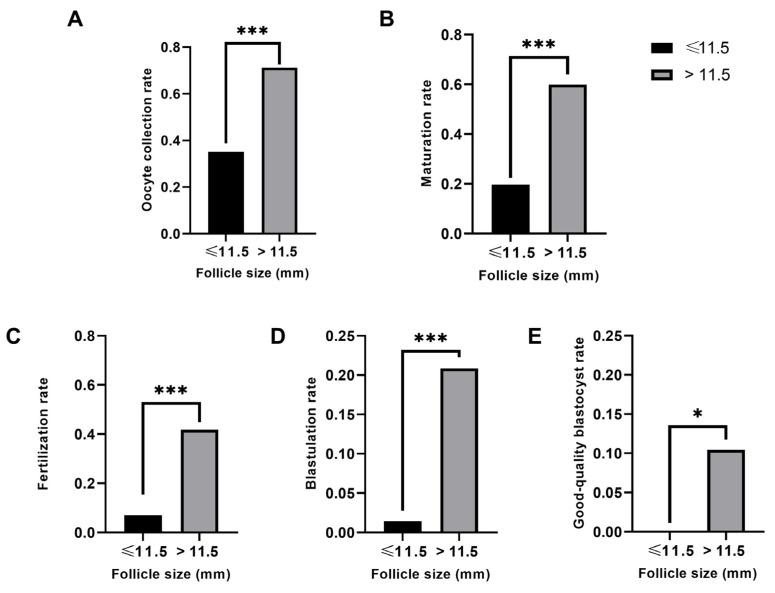
Comparison of the IVF outcomes of small (≤11.5 mm) and large (>11.5 mm) follicles. (**A**) Oocyte collection rate. (**B**) Maturation rate. (**C**) Fertilization rate. (**D**) Blastulation rate. (**E**) Good-quality blastocyst rate. All rates were calculated based on the number of punctured follicles. * *p* < 0.05. *** *p* < 0.001.

As statistically significant differences were detected in follicular sizes above and below 11.5 mm, follicles were broadly re-classified in two follicular size categories: ≤11.5 mm or >11.5 mm. This approach highlighted the significant differences with respect to follicular size, in terms of oocyte collection, maturation, fertilization, blastulation, and good-quality blastocyst rate by the number of punctured follicles (*p* < 0.01 in each case; Figure 2). None of the sixty-four follicles < 11.5 mm generated a good-quality blastocyst, whereas twelve out of one hundred and fourteen follicles > 11.5 mm did so (Table 2, Figure 2E), reinforcing the idea that a minimum follicular size of 11.5 mm is needed to obtain good-quality blastocysts.

**Figure 3:**
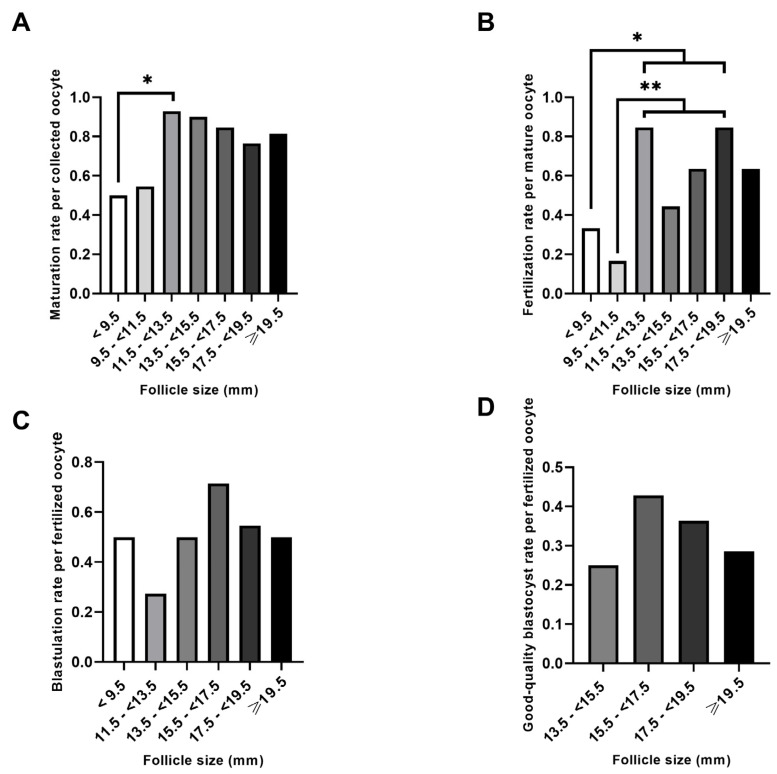
Comparison of the embryology outcomes by the number of oocytes. Rates were calculated based on the number of recovered oocytes (**A**), mature (MII) oocytes (**B**), or correctly fertilized oocytes (those with two pronuclei) (**C**,**D**). * *p* < 0.05. ** *p* < 0.01. An absence of an asterisk (*) indicates a lack of significant differences. Groups with rates of zero are not shown.

When embryology outcomes were assessed according to the number of oocytes retrieved (Figure 3A), only 11.5–<13.5 mm follicles produced significantly more MII oocytes than follicles sized < 9.5 mm (*p* < 0.05). Both follicular sizes (<9.5 mm and 9.5–<11.5 mm) were associated to significantly lower fertilization rates (relativized to the number of MII oocytes) when compared to follicles sized between 11.5–<13.5 mm and 17.5–<19.5 mm (*p* < 0.05 and *p* < 0.01, respectively; Figure 3B). Similar to the blastulation rate relativized to the number of punctured follicles, no significant differences were found in the general or good-quality blastulation rates in relation to the number of normally fertilized zygotes (Figure 3C,D).

### 2.3. An Oocyte Maturation-Related miRNA Signature Was Maintained in the Follicular Fluid of Different-Sized Follicles

**Figure 4:**
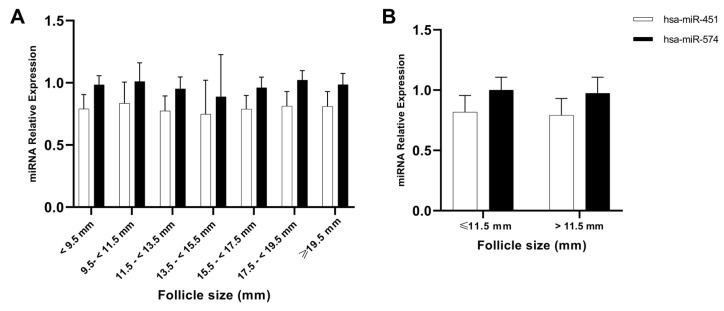
RT-qPCR evaluation of an oocyte-maturation-related miRNA signature in follicular fluid. (**A**) Relative expression of hsa-miR-451 and hsa-miR-574 in FF between the initial seven follicle groups (N (<9.5 mm) = 7, N (9.5–<11.5 mm) = 7, N (11.5–<13.5 mm) = 12, N (13.5–<15.5 mm) = 7, N (15.5–<17.5 mm) = 10, N (17.5–<19.5 mm) = 14, N (≥19.5 mm) = 19). (**B**) Relative expression of hsa-miR-451 and hsa-miR-574 in FF between follicles smaller (N = 14) or larger (N = 62) than 11.5 mm. Error bars represent standard deviation.

The expression of a previously validated oocyte-maturation-related miRNA signature (hsa-miR-451 and hsa-miR-574) [32] was assessed by real-time quantitative polymerase chain reaction (RT-qPCR) in the FF of 78 different-sized follicles. No significant differences were found in the miRNAs’ expression, with relation to the original seven follicular groups (Figure 4A) or the broader subdivision into small (≤11.5 mm) and large follicles (>11.5 mm) (Figure 4B). The fact that oocytes either from large or small follicles share the same miRNA signature associated with oocyte maturation is indicative that the oocytes will be similar in terms of nuclear competency, regardless follicular size. As shown in Figure 4, the data exhibited higher deviations when follicles were divided into seven groups due to the small sample size in each group. When follicles were reclassified into two groups based on the new cut-off value, deviations decreased as the sample size per group increased, providing more robustness to the latest results (Figure 4B).

## 3. Discussion

In clinical IVF standard practice, follicles greater than 13–14 mm are punctured for ovum pickup, while smaller follicles (<12 mm) are often disregarded [6]. This approach limits the reproductive potential of poor responders who produce a discrete number of smaller follicles in patients that sometimes do not meet the minimal requirement of at least three follicles of 17 mm or more. To address this healthcare gap, our study aimed to puncture all the developed ovarian follicles in patients with POR in order to evaluate oocyte collection, maturation, fertilization, and developmental competence in relation to follicular size.

Our findings corroborate that embryology outcomes tend to increase along with follicular size [6,8,11,12,15,24] yet support previous postulates that follicles < 16 mm still can yield good-quality embryos [7,12,15,23]. Interestingly, our found cut-off value of 11.5 mm is similar to some previously observed as 10 mm [7,12,42].

Our prospective study pointed out that, in our cohort, follicles sized 11.5 mm or larger produced mature MII oocytes according to morphological assessment, as recently described [23] and supported by the similar expression of miRNA signature associated with oocyte maturation (Figure 4). Nevertheless, none of the follicles sized < 13.5 mm yielded a blastocyst with sufficient quality for embryo transfer or cryopreservation, which corroborates previous evidence that good-quality blastocysts are only derived from follicles with diameters of minimum 12.5 mm [8,23]. In alignment with our results, follicles sized as 11.5 mm, which are often disregarded during oocyte retrieval, produce mature MII oocytes, meaning that oocytes derived from these follicles have achieved nuclear competence. This is a key finding in patients with a limited number of follicles considering that the retrieval of oocytes from follicles sized 11.5 mm and above lead to an increase in the number of MII oocytes available for IVF [9,25]. Unfortunately, it seems that nuclear competence is not sufficient to improve IVF outcomes. This can be owed to the fact that full oocyte competence requires not only nuclear competence exhibited by MII oocytes but also cytoplasmatic competence. Indeed, fine-tuning the coupling between nuclear and cytoplasmic maturation in the context of in vitro maturation (IVM) cycles has improved treatment efficacy [43]. The lack of fully developmental competence of mature oocytes evinced in our study reveals that these oocytes are not cytoplasmic competent. This means that these not fully competent oocytes are not able to sustain chromosomal rearrangements, epigenetic modifications, the complex process of fertilization, and the subsequent cleavage and embryo development [44].

The oocyte’s quality or capacity to resume meiosis and acquire complete developmental competence depends on the bidirectional crosstalk between the granulosa cells and the oocyte during folliculogenesis, as follicular diameter increases together with developmental capacity. At the antral follicle stage, oocytes should be able to resume meiosis (i.e., achieve nuclear maturation), and this ability is closely related to the size of the oocyte [45]. On the contrary, cytoplasmic competence is measured by the oocyte’s ability to be fertilized and develop into a blastocyst by the time of ovulation [46]. Based on these findings, an impaired synchrony of nuclear and cytoplasmic maturation may explain why oocytes obtained from follicles sized < 11.5 mm had lower fertilization rates [11,12,14,15,21,22,23]. For this reason, the mature oocytes derived from follicles sized < 11.5 mm might be suitable candidates for IVM protocols aimed to improve oocyte quality and competence [47]. In fact, oocytes derived from follicles smaller than 10 mm that have undergone IVM as well as pre-IVM preparation reported and enhanced rate of good-quality blastocysts [43,48,49].

The number of oocytes retrieved positively correlated with reproductive outcomes such as the number of correctly fertilized oocytes [50], blastocysts obtained per stimulation cycle [50,51], and proportion of good-quality embryos [51]. While 13 oocytes have been thought to be sufficient to produce high pregnancy rates, both per transfer and per initiated stimulation cycle [52], other studies agreed on the fact that achieving 20 oocytes per cycle results in the highest primary and cumulative live birth rates [50,53]. Although these data reinforce the importance of maximizing ovum pickup by aspirating all antral follicles in poor responders to optimize their reproductive success, the authors note that the current ovum pickup methodology needs additional refinement to enhance the oocyte collection efficiency in follicles < 11.5 mm in diameter.

Our results highlight the untapped potential of follicles larger than 11.5 mm in patients with POR described recently [23] by demonstrating that nearly 70% of follicles larger than 11.5 mm contain an oocyte, and developmentally competent oocytes can be recovered from follicles that are minimum 11.5 mm in diameter.

A miRNA signature associated with oocyte maturation (hsa-miR-451 and hsa-miR-574) in FF, previously published by our group [41], was used here to investigate possible differences in oocyte maturation rate among different follicular size groups. Mature oocytes show hsa-miR-451 downregulation and hsa-miR-574 upregulation [41]. However, uniform miRNA expression across follicular groups confirmed the presence of mature oocytes, which was an unexpected finding considering the significant differences in oocyte maturation rates found between follicles < 9.5 mm and 11.5–<13.5 mm (Figure 1B and Figure 3B) or above and below the 11.5 mm threshold (Figure 2B). Thus, a more in-depth molecular characterization of oocytes from smaller follicles should be considered to cross-validate these findings in larger cohorts of POR patients undergoing IVF.

## 4. Materials and Methods

### 4.1. Study Population

Thirty-one patients with POR, undergoing IVF and posterior embryo transfer, were recruited between 2019 and 2021 at the IVIRMA Valencia clinic (Valencia, Spain). All participants were aged 29–39 years old and reported a median AMH level of 0.47 ng/dL [IQR: 0.27–0.83] and a BMI below 30 Kg/m^2^ (Table 1). All patients were expected to have a reduced ovarian response according to POSEIDON criteria [5] (Groups 3 and 4) and had a low ovarian response in previous stimulation with conventional stimulation. All recruited patients had a maximum total number of 5 follicles (with at least one of them being <18 mm in size) on the day of ovulation trigger. In the IVF cycles considered for the current research, all patients received mild or minimal ovarian stimulation. The minimal ovarian stimulation was followed using anti-estrogens (clomiphene citrate) throughout the stimulation cycle and a dose of 150 IU human menopausal gonadotrophin (hMG) or follicle-stimulating hormone (FSH) plus luteinizing hormone (LH) every other day from day 4 of treatment [42]. The American Society for Reproductive Medicine (ASRM) recommends the possibility of using mild stimulation protocols in expected POR patients (Groups 3 and 4 in POSEIDON criteria), underlying the fact that clinical pregnancy rates after conventional IVF gonadotropin protocols are similar to those obtained after mild ovarian stimulation protocols using low-dose gonadotropins (<150 IU/day) [54]. The use of a mild ovarian stimulation strategy in cases of POR provides several benefits, including better patient comfort; a decrease in both the duration and dosage of gonadotropins; and, therefore, an overall reduction in the cost per cycle of ovarian stimulation [55]. Women were excluded from the study if they were undergoing luteal phase and long agonist stimulation protocols, presented with endometriosis, abnormal karyotypes, or cycles involving the use of non-ejaculated spermatozoa.

### 4.2. Study Design

In total, 178 follicles from 31 patients were individually punctured during transvaginal oocyte retrieval. To avoid cross-contamination, the tubing of the aspiration system was flushed prior to each follicle puncture. Follicles were classified into seven groups according to their diameter (<9.5 mm, 9.5–<11.5 mm, 11.5–<13.5 mm, 13.5–<15.5 mm, 15.5–<17.5 mm, 17.5–<19.5 mm, and ≥19.5 mm). Oocyte collection, maturation, fertilization, blastulation, and good-quality blastocyst rates were recorded for each follicular-size-based group. Individual FF aspirates were processed as previously described [41]. The 1 mL aliquots of FF were stored at −80 °C until RT-qPCR analysis to evaluate the presence of an oocyte-maturation-related miRNA signature previously validated by our group [41].

### 4.3. miRNA Isolation from Follicular Fluid and Real-Time Quantitative Polymerase Chain Reaction (RT-qPCR)

MiRNAs were isolated from 200 µL of FF using the miRNeasy Serum/Plasma Kit (Qiagen, 217184, Hilden, Germany), following the manufacturer’s recommendations. RNA quantity (ng/mL) and quality (260/280 and 260/230 ratios) were measured in a NanoDrop 2000 spectrophotometer system (Thermo-Scientific, Tewksbury, MA, USA). MiRNA-specific cDNA synthesis was performed using the miRCURY LNA RT Kit (Qiagen, 339340, Hilden, Germany). The specific primer assays for hsa-miR-451, hsa-miR-574, two constitutively expressed miRNA targets (hsa-miR-103a and hsa-miR-191; positive controls), and UniSP6 (internal control) were designed and commercially synthesized by LNA Technology (Qiagen, Hilden, Germany) (Supplementary Table S1). RT-qPCR was performed using the miRCURY LNA SYBR Green PCR Kit (Qiagen, 339346) on a StepOnePlus RT-PCR system (Applied Biosystems, Woburn, MA, USA) with the following conditions: 95 °C 2 min for initial heat activation and 40 cycles of denaturation (95 °C 10 s), followed by annealing (56 °C 60 s). Differential expression of hsa-miR-451 and hsa-miR-574 was carried out by comparing the normalized cycle threshold (Ct) values for all biological replicates.

### 4.4. Assessment of Clinical Outcomes

Follicular size was measured via transvaginal ultrasound, on the day of oocyte retrieval, and was estimated as the mean of the longest diameters (mm) in perpendicular planes. The presence of an oocyte within the aspirate of each follicle was recorded. Oocyte maturation was evaluated during the cumulus–oocyte complex denudation. Oocytes were considered to be germinal vesicle (prophase I); metaphase I; or MII if there was a single, extruded polar body. ICSI was performed in all cases, regardless of sperm parameters, to standardize the fertilization method. Approximately 18 h after ICSI, the number of normally fertilized oocytes was determined by the presence of two adjacent pronuclei. Embryos were cultured in vitro until they reached the blastocyst stage on days 5–6. The ASEBIR score was used for blastocyst grading [56]. Notably, this study used the ovarian follicle, rather than the oocyte derived from the follicle, as the primary unit of analysis to assess the efficiency of oocyte collection (the number of oocytes collected from all the punctured follicles) [8] and relationships between follicular sizes and IVF outcomes. In this regard, the maturation, fertilization, and blastulation rates were first calculated per punctured follicle (Figure 1 and Figure 2, Table 2), then in relation to the number of MII oocytes or fertilized zygotes, respectively (Figure 3, Table 2).

### 4.5. Statistical Analyses

Participants’ baseline and reproductive characteristics were presented as mean ± standard deviations or median [interquartile range] according to their distribution. The Kolmogorov–Smirnov test was chosen to evaluate distribution due to the small sample size. Clinical outcomes were compared for each follicle group. Chi-squared analyses were used to compare the oocyte collection rates among groups. Due to the limited sample size, Fisher’s exact tests were used to compare rates of oocyte maturation, fertilization, blastulation, and good-quality blastocysts among groups in pairs. Moreover, the *t*-test and Mann–Whitney U test were used depending on normality test when follicles were divided in two groups. MiRNA data were presented as mean ± standard deviations and analyzed with one-way ANOVA or Kruskal–Wallis test depending on normality test result. In all cases, *p* < 0.05 was considered statistically significant. All these analyses were performed using GraphPad Prism version 8.3.0 for Windows (GraphPad Software, Boston, Massachusetts, USA).

## 5. Conclusions

The majority of oocytes recovered from follicles smaller than 17.5 mm can be useful in IVF cycles of POR patients. Specifically, follicles as small as 11.5 mm can produce mature oocytes but only those larger than 13.5 mm gave rise to a transferable embryo. Further, if combined with additional protocols aiming to complete cytoplasmic maturation in follicles smaller than 11.5 mm in size, this would be a promising strategy for maximizing the likelihood of reproductive success in these patients. In this way, smaller follicles can maximize the number of mature oocytes retrieved per stimulation cycle in patients reporting POR.

Our data corroborates previous evidence that good-quality blastocysts are only derived from follicles with diameters above 13.5 mm. These findings confirm the IVF standard practice of follicular puncture is adequate.

In addition to the stable expression of oocyte-maturation-related miRNA (hsa-miR-451 and hsa-miR-574) validating the maturation of the collected oocytes, this suggest that these factors may not be relevant in identifying the best oocytes. However, a more in-depth molecular characterization of the oocytes derived from smaller follicles is recommended.

Nevertheless, all these data may help clinical decision making and avoid cancelling stimulation cycles when poor responders do not meet standard criteria for triggering ovulation. 

## Figures and Tables

**Table 1:** Baseline reproductive characteristics of the patients (n = 31).

**Demographic Characteristics**	
Age (years), median [IQR]	37 [35–38]
Weight (kg), mean (SD)	59.02 (8.81)
Height (m), mean (SD)	1.637 (0.072)
BMI (kg/m^2^), mean (SD)	22.04 (3.13)
**Reproductive Characteristics**	
Stimulation time (days), mean (SD)	11.28 (2.87)
Total gonadotropin dose (IU), mean (SD)	2042 (957.1)
Serum E2 on trigger day (pg/mL), mean (SD)	952.3 (392.3)
Serum P4 on trigger day (ng/mL), mean (SD)	0.583 (0.378)
Serum AMH (ng/mL), median [IQR]	0.47 [0.27–0.83]

Abbreviations: IQR, interquartile range; BMI, body mass index; E2, estradiol; P4, progesterone; AMH, anti-Müllerin hormone.

**Table 2:** Embryology outcomes by follicular-size-based groups.

Follicular Diameter (mm)	Punctured Follicles (n)	Oocytes		Mature Oocytes (MII)			Fertilized Oocytes (2PN)			Blastocysts			Good-Quality Blastocysts		
		n	PPF	n	PPF	Per Oocyte	n	PPF	Per MII Oocyte	n	PPF	Per 2PN Oocyte	n	PPF	Per 2PN Oocyte
<9.5	43	12	27.91%	6	13.95%	50.00%	2	4.65%	33.33%	1	2.33%	50.00%	0	0.00%	0.00%
9.5–<11.5	21	11	52.38%	6	28.57%	54.55%	1	4.76%	16.67%	0	0.00%	0.00%	0	0.00%	0.00%
11.5–<13.5	21	14	66.67%	13	61.90%	92.86%	11	52.38%	84.62%	3	14.29%	27.27%	0	0.00%	0.00%
13.5–<15.5	16	10	62.50%	9	56.25%	90.00%	4	25.00%	44.44%	2	12.50%	50.00%	1	6.25%	25.00%
15.5–<17.5	15	13	86.67%	11	73.33%	84.62%	7	46.67%	63.64%	5	33.33%	71.43%	3	20.00%	42.86%
17.5–19.5	23	17	73.91%	13	56.52%	76.47%	11	47.83%	84.62%	6	26.09%	54.55%	4	17.39%	36.36%
≥19.5	39	27	69.23%	22	56.41%	81.48%	14	35.90%	63.64%	7	17.95%	50.00%	4	10.26%	28.57%
Overall	178	104	58.43%	80	44.94%	76.92%	50	28.09%	62.50%	24	13.48%	48.00%	12	6.74%	24.00%

Follicles were classified into seven groups according to their size. The rates of oocyte collection, maturation, fertilization, blastulation, and good-quality blastocysts were calculated relative to the number of punctured follicles and recovered, mature, or fertilized oocytes. Abbreviations: PPF, per punctured follicle; MII, metaphase II; 2PN, two pronuclei.

## Data Availability

All data generated or analyzed during this study are included in this published article and its Supplementary Information Files.

## Supplementary Materials

The following supporting information can be downloaded at https://doi.org/10.17632/sj5kbg68cd.1 (accessed on 23 September 2024).
